# Antibacterial and Immunomodulatory Properties of Acellular Wharton’s Jelly Matrix

**DOI:** 10.3390/biomedicines10020227

**Published:** 2022-01-21

**Authors:** Marie Dubus, Loïc Scomazzon, Julie Chevrier, Charlotte Ledouble, Adrien Baldit, Julien Braux, Florelle Gindraux, Camille Boulagnon, Sandra Audonnet, Marius Colin, Hassan Rammal, Cédric Mauprivez, Halima Kerdjoudj

**Affiliations:** 1 Biomatériaux et Inflammation en Site Osseux (BIOS) EA 4691, Université de Reims Champagne Ardenne, 51100 Reims, France; loic.scomazzon@univ-reims.fr (L.S.); julie.chevrier@univ-reims.fr (J.C.); charlotte.ledouble@univ-reims.fr (C.L.); julien.braux@univ-reims.fr (J.B.); marius.colin@univ-reims.fr (M.C.); hkrammal@hotmail.com (H.R.); cedric.mauprivez@univ-reims.fr (C.M.); 2 Dentistry Faculty, Université de Reims Champagne Ardenne, 51100 Reims, France; 3 Pôle Médecine Bucco-Dentaire, Hôpital Maison Blanche, Centre Hospitalier Universitaire de Reims, 51100 Reims, France; 4 Laboratoire d’Étude des Microstructures et de Mécanique des Matériaux (LEM3)—UMR CNRS 7239, Université de Lorraine, 57070 Metz, France; adrien.baldit@univ-lorraine.fr; 5 Laboratoire de Nanomédecine, Imagerie, Thérapeutique EA 4662, Université Bourgogne Franche-Comté, 25000 Besançon, France; f.gindraux@chu-besancon.fr; 6 Laboratoire D’anatomie et Cytologie Pathologique, Centre Hospitalo-Universitaire, Hôpital Robert Debré, 51100 Reims, France; camille.boulagnon@univ-reims.fr; 7 Plateau Technique URCACyt, Université de Reims Champagne Ardenne, 51100 Reims, France; sandra.audonnet@univ-reims.fr; 8 Pharmacy Faculty, Université de Reims Champagne Ardenne, 51100 Reims, France

**Keywords:** Wharton’s jelly, decellularization, bioactivity, antibacterial, immunomodulation

## Abstract

Of all biologic matrices, decellularized tissues have emerged as a promising tool in the field of regenerative medicine. Few empirical clinical studies have shown that Wharton’s jelly (WJ) of the human umbilical cord promotes wound closure and reduces wound-related infections. In this scope, we herein investigated whether decellularized (DC)-WJ could be used as an engineered biomaterial. In comparison with devitalized (DV)-WJ, our results showed an inherent effect of DC-WJ on Gram positive (*S. aureus* and *S. epidermidis*) and Gram negative (*E. coli* and *P. aeruginosa*) growth and adhesion. Although DC-WJ activated the neutrophils and monocytes in a comparable magnitude to DV-WJ, macrophages modulated their phenotypes and polarization states from the resting M0 phenotype to the hybrid M1/M2 phenotype in the presence of DC-WJ. M1 phenotype was predominant in the presence of DV-WJ. Finally, the subcutaneous implantation of DC-WJ showed total resorption after three weeks of implantation without any sign of foreign body reaction. These significant data shed light on the potential regenerative application of DC-WJ in providing a suitable biomaterial for tissue regenerative medicine and an ideal strategy to prevent wound-associated infections.

## 1. Introduction

Tissue damages are currently one of the biggest health issues in the world. Arising from disease or trauma, a complete treatment requires the reparation of the injured site. Autografts are often considered as the “gold standard”, however, inherent limitations including donor site morbidity, low availability, and elevated failure rates highlight the need for alternative strategies. Conventional practices use allografts but the deficit between donors and patients requiring the graft has grown in recent years, attracting huge interest in the regenerative medicine field. 

Over the past two decades, regenerative medicine has emerged with the aim of directly repairing and regenerating damaged tissues, alleviating the necessity for tissue donation. In this context, several scaffolds have been developed [1,2] and most of the studies have proven the superiority of biological scaffolds to synthetic ones [3,4,5]. Indeed, by using a naturally derived extracellular matrix (ECM) that recapitulates the hierarchical complexity of native tissues, it is possible to mimic the structural, biochemical, and mechanical cues suitable for the recruitment of endogenous cells, inducing therefore the tissue regeneration instead of scarring. In this scenario, biological scaffolds could be obtained through the process of devitalization or decellularization, providing reliable stocks for the regenerative medicine demands [6,7,8,9,10,11,12]. Devitalization, obtained by physical processing such as freeze–thaw cycles or freezer-milling, disrupts cellular and nuclear membranes but may not fully remove DNA moieties, cell-associated proteins, and other cell remnants [10]. In addition to a potential immune rejection risk, a limited porosity of devitalized tissues poses a challenge for cell infiltration and tissue integration [13,14,15]. Therefore, the ultimate goal of decellularization is to rid the ECM of cells and genetic materials, limiting the immune risk [11,12,16,17]. An increase in pore size along with a reduction in the mechanical performance of the resulting decellularized scaffolds was, however, reported [17].

Perinatal tissues (i.e., fetal membranes and umbilical cord) represent a valuable opportunity for the development of biological scaffolds as they can be easily achieved, both from a technical and an ethical point of view. Clinically, perinatal tissues have been used as allografts across multiple disciplines including ocular surface reconstruction, burn and ulcer treatments, and craniofacial reconstruction [18,19,20,21,22,23]. While the clinical use of fetal membranes is well documented, the use of umbilical cord tissue in this setting is relatively new. Umbilical cord-derived Wharton’s jelly (WJ) is a mucous connective tissue that is covered by a layer of amniotic epithelium and surrounds the umbilical blood vessels. The WJ matrix, mainly composed of glycosaminoglycans (GAGs) and collagen fibers, acts to prevent compression of the vessels [24,25,26,27,28]. The hydrated and viscous state of WJ is attributed to the high percentage of high molecular weight hyaluronic acid content, making WJ matrix an attractive option for regenerative medicine and matrix-based therapy [20,29]. In this context, substantial empirical clinical reports emphasize the regenerative therapeutic potential of devitalized umbilical cord for skin repair defect associated with bone exposure in medically fragile patients (i.e., diabetes, ischemia and osteomyelitis). The devitalized umbilical cord matrix therapy is associated with an overall healing rate from 79 to 100% versus 32% under a standard of care [30,31,32,33]. These encouraging results are mainly attributed to the presence of bioactive components in clinically relevant quantities compared with other biologics [20,25,34].

Decellularized and devitalized biological scaffolds are attractive tools as they are custom-made by the endogenous residing cells, however, research highlighting the host immune response towards these scaffolds is limited. Following the implantation of biomaterials, a suitable inflammatory stimulation is required for wound healing and tissue regeneration; but an excessive and disproportionate inflammatory reaction leads to adverse events, with resultant scarring and encapsulation [35,36]. Therefore, the understanding of the host’s immune response caused by the foreign WJ-derived scaffold is the key to seeking the potential strategy to regenerate damaged tissues. In the aim of choosing the most successful pathway for the development of WJ-derived matrix, the present study seeks to settle if full decellularization is desired or if devitalization is acceptable. Herein, we developed a reliable method for the total decellularization of WJ. Changes in the structural, biochemical and biomechanical properties of the resulting matrix were firstly investigated. The biological performance of both decellularized (DC)-WJ and devitalized (DV)-WJ was assessed taking into consideration the (i) cytotoxicity, (ii) antibacterial properties, (iii) innate immune response towards neutrophils and monocytes, (iv) immunomodulatory effect on macrophage polarization and (v) in vivo response following the subcutaneous implantation and calvaria bone defect regeneration.

## 2. Materials and Methods

All human sample harvesting was approved ethically and methodologically by our local Research Institution and was conducted with informed patients (written consent) in accordance with the usual ethical legal regulations (Article R 1243-57). All procedures were performed in accordance with our authorization and registration number DC-2014-2262 given by the National “Cellule de Bioéthique”. 

### 2.1. Preparation of Samples 

Fresh human umbilical cords, obtained after full-term births, were washed several times with Phosphate Buffered Saline (PBS, Gibco, Villebon-sur-Yvette, France) to remove blood components, dissected and vascular structures removed. Wharton’s jelly matrix (WJ) was then peeled off the amniotic surrounding membrane, and preserved at −20 °C. After two cycles of freezing/defrosting (−20 °C/20 °C), devitalized samples were subjected to a decellularization protocol comprising hypotonic treatment with 1% Triton X-100 in distilled water (VWR, Rosny-sous-Bois, France) for 1 h then enzymatic treatment with 0.2 mg/mL of DNase (Sigma, Saint-Quentin Fallavier, France) at 37 °C for 24 h under stirring. All processing residuals were removed by rinsing twice with PBS for 10 min under stirring. Finally, decellularized Wharton’s jelly (DC-WJ) samples were frozen at −20 °C then −80 °C before the freeze-drying process. Freeze-dried devitalized WJ (DV-WJ) was used as a control.

### 2.2. Matrix Characterization 

The efficiency of the decellularization protocol was firstly verified by nuclei staining with 4,6-diamidino-2-phenylindole (DAPI, 100 ng/mL, 1:3000 dilution) for 5 min and fluorescence microscopy imaging (Axiovert 200 M microscope, Zeiss, Oberkochen, Germany, Objective × 10). Secondly, DNA was extracted from samples using the MasterPure^TM^ DNA Purification Kit (Epicentre^®^ Biotechnologies, Euromedex, Souffelweyersheim, France) in accordance with the manufacturer’s protocol. Freeze-dried samples were weighed prior to DNA extraction. Extracted DNA was then assessed by measuring the absorbance at 260 and 280 nm (Nanodrop^®^, Thermo Scientific, Waltham, MA, USA)) with a 260/280 nm absorbance ratio for all measured samples comprised between 1.8 and 2. DNA concentration was calculated according to tissue weight (ng of DNA/µL/mg of dry tissue). Finally, samples were embedded in paraffin and cut into 4 μm sections (rotation microtome RM2055, Leica Microsystems, Nanterre, France). Hematoxylin-Eosin-Saffron (HES) staining was performed, and images were taken using vs. 120 OLYMPUS scanner. 

The integrity of the extracellular matrix was assessed by using colorimetric assays. To this end, a colorimetric total collagen assay kit (BioVision, Nanterre, France), and Alcian Blue 8GX (Sigma, Saint-Quentin Fallavier, France) in HCl 0.1 M (pH 1.5 or pH 2.5) were used. Briefly, hydrolyzed samples (15 µL) were mixed with Chloramine T (100 µL) and incubated for 5 min before adding p-dimethylaminobenzaldehyde reagent (100 µL). After 90 min of incubation at 60 °C, the absorbance was measured at 560 nm (FLUOstar Omega microplate reader, BMG Labtech) and the total collagen concentration, calculated using a kit standard curve, was normalized according to the weight of the dry sample (mg of collagen/mg of dry tissue). Sulfated and non-sulfated glycosaminoglycans (GAGs) were quantified at pH 1.5 and 2.5, by adding 1 mL of 0.5% (*w*/*v*) Alcian Blue 8GX to samples and incubation overnight on an orbital shaker (300 rpm). After supernatant removal, fixed Alcian Blue was dissolved in HCl 6 M and the absorbance was measured at 600 nm. Concentrations of sulfated and non-sulfated GAGs were determined, respectively, according to chondroitin sulfate B sodium (Sigma, Saint-Quentin Fallavier, France) and hyaluronic acid (RenovHyal^®^, Givaudan, Pomacle, France) standard range, were normalized according to the weight of the dry sample (µg of GAG/mg of dry tissue).

The release of macromolecules and growth factors from samples (soaked in 1 mL of FBS-free α-MEM or in 1 mL of α- MEM supplemented with 10% of FBS culture media for 72 h) was respectively determined using Autoflex speed MALDI-TOF/TOF mass spectrometer (Bruker, Palaiseau, France) and ELISA kits (i.e., Human Duoset^®^ VEGF, HGF and TGF-β (R&D systems, Lille, France)), according to the manufacturer’s instructions.

Structural characterizations of freeze-dried samples were conducted using scanning electron microscopy with a field emission gun (FEG-SEM, JEOL JSM-7900F, Croissy, France) and two-photon excitation laser scanning confocal microscopy and second harmonic generation (SHG; LSM 710-NLO, Carl Zeiss SAS, Rueil-Malmaison, France) coupled with CHAMELEON femtosecond Titanium-Sapphire Laser. FEG-SEM images were acquired from secondary electrons at primary beam energy at 5 kV. Samples were excited at 860 nm and SHG signal was collected under circular polarization in 420–440 nm spectral window with ×20 objective (ON: 0.8). The total porosity of samples was assessed by mercury intrusion porosimetry (Micromeretics AutoPore IV 9500, Hexton, UK). The swelling ratio of samples was determined by a fluid absorption method and the equilibrium swelling ratio (*Q*) was calculated using the following equation:

(1) $$Q=\frac{\left(Wwet-Wdry\right)}{Wdry}$$

where “*W dry*” corresponds to the weight of the freeze-dried sample while “*W wet*” corresponds to the weight of the PBS hydrated sample.

Finally, the mechanical properties of samples were tested through quasi-static tensile tests up to failure using Universal Testing Machine Zwicky 0.5 equipped with a 10 N loadcell. 

### 2.3. Biocompatibility 

#### 2.3.1. Cell Culture

In this study, human gingival-derived fibroblasts (Fibro) and human mandibular pre-osteoblasts (Osteo) were used for in vitro studies. Fibro were isolated from gingival fragments, obtained during teeth removal. Osteo were isolated from the mandibular bone without any clinical or radiographic evidence of pathology from young patients (aged 13–33 years) undergoing windows teeth extraction oral surgery. Fibro and Osteo were cultured in DMEM-Glutamax^®^ (Gibco, Villebon-sur-Yvette, France) supplemented with 10% heat-inactivated (30 min, 56 °C) fetal bovine serum (FBS) and 1% Penicillin/Streptomycin (PS, Gibco, Villebon-sur-Yvette, France).

#### 2.3.2. Cytotoxicity Tests

Fibro and Osteo were seeded at 3 × 10^4^ cells/cm^2^ in a 96 well culture plate for 24 h, then 1 mg of UV-decontaminated (30 min) freeze-dried samples were added in culture wells. Fibro and Osteo cultured on tissue culture plastic without any sample were used as controls. According to the ISO/EN 10,993 part 5 guideline, the cytotoxicity of sample released agents was monitored after 24 h of contact with cells by WST-1^®^ (water-soluble tetrazolium salt-1^®^, Roche Diagnostics, Meylan, France) assay and in culture supernatants by LDH (lactate dehydrogenase, Roche Diagnostics, Meylan, France), in accordance with the manufacturer protocol. Absorbances were measured at 440 nm (with a correction wavelength at 750 nm, for WST-1^®^) and at 492 nm (with a correction wavelength at 700 nm, for LDH) using a FLUOstar Omega microplate reader (BMG Labtech, Champigny-sur-Marne, France). 

For Fibro and Osteo optical microscopy observation in contact of samples, 10^4^ cells were seeded on 5 mm-diameter UV-decontaminated samples glued (R41 wood vinylic glue, BOSTIK^®^, Colombes, France) in 96 well culture plates. After seven days of culture, cells were stained with crystal violet and imaged with an EVOS^®^ digital microscope.

#### 2.3.3. Chemotaxis Assay

The chemotactic activity of samples on Fibro and Osteo was investigated through transwell cell migration assay. 1 mg of UV-decontaminated samples were deposited in 24 well culture plates in 500 µL of serum-free cell culture medium, whereas 5 × 10^3^ of Fibro or Osteo were seeded on the top of a cell culture insert membrane (Millicell^®^ Hanging Cell Culture Inserts, Merck Millipore, Guyancourt, France). After 48 h of incubation at 37 °C in 5% CO_2_, non-migrating cells were removed from the top of the membrane and migrated cells at the bottom were fixed with methanol (5 min) then stained with crystal violet (1 h). Migrated cells were imaged with an EVOS^®^ digital microscope and counted. A serum-free cell culture medium without any sample was used as a control.

### 2.4. Antibacterial Activities

For all bacterial experiments, *Staphylococcus aureus* (*S. aureus*, SH1000), *Staphylococcus epidermidis* (*S. epidermidis*, CIP 53.124), *Pseudomonas aeruginosa* (*P. aeruginosa*, ATCC 9027) and *Escherichia coli* (*E. coli*, CIP 54.8 T) were grown on Trypto-casein Soy (TCS) agars (Biokar) at 37 °C. All strains were then grown for 18 h in nutritive broth at 37 °C and absorbances of each culture at 600 nm were adjusted to 1 before dilution at 1/500 in nutritive broth.

#### 2.4.1. Planktonic Growth and Bacteria Adhesion

1 mL of bacterial cultures were deposited on UV-decontaminated freeze-dried samples placed into 24 well culture plates. After 24 h of culture at 37 °C, samples were removed for bacteria adhesion experiments and absorbances were measured at 600 nm. For bacteria adhesion experiments, samples were rinsed with nutritive broth, immersed in 2 mL of nutritive broth and then sonicated for 5 min (40 kHz). Serial dilutions were further plated on TCS agars plates, and the rate of viable adhered bacteria was determined after colony count.

#### 2.4.2. Scanning Electron Microscopy

After 24 h of culture, samples were rinsed with PBS and fixed in 2.5% glutaraldehyde (Sigma) for 1 h, dehydrated in graded ethanol solutions for 10 min each and desiccated in hexamethyldisilazane (Sigma). After air-drying at room temperature, samples were sputtered with a thin gold-palladium film (JEOL ion sputter JFC 1100, Croissy Sur Seine, France). Adhered bacteria were observed using FEG-SEM (JEOL-JSM-7900F). 

#### 2.4.3. Confocal Laser Scanning Microscopy

For the visualization of alive and adhered bacteria on samples, bacteria were labeled with Syto 9 fluorescent dye and propidium iodine (Thermo Fischer, Villebon-sur-Yvette, France) for 30 min. Bacteria were then imaged by confocal laser scanning microscopy (CLSM, LSM 710 NLO, ×20 objective, Numerical Aperture 1.4, Zeiss, Oberkochen, Germany).

### 2.5. Acute Inflammatory Response 

Fresh human venous blood was collected in BD Vacutainer^®^ K2E EDTA tubes from healthy donors. Neutrophils and monocytes were isolated from whole blood by using a density gradient centrifugation medium (v/v, Granulosep, Eurobio-Abcys, Les Ulis, France). For neutrophil collection, residual erythrocytes were removed by a hypotonic shock and the resulting neutrophils were cultured in RPMI 1640-Glutamax^®^ supplemented with 2.5% heat-inactivated autologous human serum. For monocytes collection, cells were purified by a positive selection with CD14 immunomagnetic beads (MACS, Miltenyi Biotec, Paris, France), according to the manufacturer’s instructions. The resulting monocytes were cultured in RPMI 1640-Glutamax^®^ supplemented with 2.5% heat-inactivated FBS and 1% Penicillin/Streptomycin.

#### 2.5.1. Innate Neutrophil Response

Neutrophils were seeded at 10^6^ cells/mL in a 24 well culture plate in the presence of UV-decontaminated freeze-dried samples (±Lipopolysaccharide (LPS), 10 ng/mL, *E. coli* 0111:B4, Sigma). After 2 h of incubation at 37 °C and 5% CO_2_, neutrophils were labeled using Muse oxidative stress kit (Luminex) following the manufacturer’s recommendation, and intracellular ROS accumulation was analyzed by flow cytometry (LSRFortessa BD Biosciences, San Jose, CA, USA). Supernatants were collected and interleukine-8 (IL-8) release was measured using human Duoset^®^ IL-8 (R&D systems, Lille, France) according to the manufacturer’s instructions. Absorbance was measured at 450 nm. Phagocytosis capacity of neutrophils in the presence of conditioned media (from samples soaked in 1 mL of α- MEM supplemented with 10% of heat-inactivated FBS culture medium for 72 h) was established using pHrodo red *S. aureus* BioParticles conjugate (Invitrogen). Neutrophils (2.5 × 10^5^) and conditioned media (*v*/*v*) were incubated with 10 µL of pHrodo beads (containing 2.5 × 10^5^ particles) for 30 min at 37 °C and 5% CO_2_, before analyzing by flow cytometry.

#### 2.5.2. Innate Monocyte Response

CD14 positive monocytes were seeded at 5 × 10^5^ cells/mL in a 24 well culture plate in the presence of UV-decontaminated freeze-dried samples and incubated at 37 °C and 5% CO_2_ for 4 h and 24 h. Supernatants were collected and Tumor necrosis factor-alpha (TNF-α), interleukine-1 beta (IL-1β), and interleukine-10 (IL-10) release was measured using human Duoset^®^ TNF-α, IL-1β and IL10 (R&D systems), according to manufacturer’s instructions. absorbances were measured at 450 nm. LPS (10 ng/mL) was used as an inflammatory stimulus control.

### 2.6. Immunomodulation

THP-1 pro-monocytic cell line was purchased from the American Type Culture Collection. Cells were cultured at a density of 2 × 10^5^ cells/mL in a 75 cm^2^ Flask in RPMI 1640 medium supplemented with 10% FBS, 1% PS and maintained in a humidified atmosphere of 5% CO_2_ at 37 °C with a medium change every 2 days. THP-1 cells were seeded at 10^6^ cells/mL in 24 well plates and treated with 80 mM phorbol 12-myristate 13-acetate (PMA, Thermo Fischer, Villebon-sur-Yvette, France) for 24 h. Following PMA treatment, cells were washed with PBS and fresh RPMI medium supplemented with 5% FBS, 1% PS was added onto the THP-differentiated macrophage (M0) for 24 h. UV-decontaminated freeze-dried samples were then added in each well, as well as fresh RPMI medium, for 48 h or 72 h. Controls for pro-inflammatory (M1) and anti-inflammatory (M2) phenotypes were made by treating M0 macrophages with 20 ng/mL IFN-γ (R&D systems) and 100 ng/mL LPS for M1 phenotype, and 20 ng/mL IL-4 (R&D systems) for M2 phenotype, for 48 h or 72 h. After treatment, supernatants were collected and stored at −20 °C for cytokine production measurements. TNF-α, IL-1β, IL-10, VEGF and TGF-β release were measured as previously described. Interleukine-1 receptor antagonist (IL-1ra) release was measured using human Duoset^®^ IL-1ra (R&D systems), according to the manufacturer’s instructions. 

### 2.7. In Vivo Evaluations 

In vivo animal tests were carried out following the guidelines approved by the Committee on Animal Care of Bourgogne Franche-Comté University (N° 2010-2206-02314) (Subcutaneous implantation) or of Reims University (N°2018111612178592) (bone calvaria defect). Animals were anesthetized through inhalation of 4% sevoflurane (Isuflu-Vet, Voorshofen, The Netherland) and euthanized at the end of each procedure by cervical dislocation with an overdose of Pentobarbital (Dolethal, Vétoquinol, France). 

#### 2.7.1. Subcutaneous Implantation

In vivo biological response to decellularized Wharton’s jelly membranes was assessed with immunocompetent eight week old male Wistar rats (*n* = 4). UV-decontaminated (30 min) samples were cut with a scalpel into some pieces weighing around 10 mg. The resulting membranes were subcutaneously sutured, and each rat received two samples (according to 3R rules). After three weeks of implantation, rats were sacrificed and the implanted membranes, the surrounding connective tissues and skin were resected from the underlying muscles. Samples were fixed in 4% paraformaldehyde, embedded in paraffin and cut into 4 μm sections as previously described. HES and Masson Trichrome (MT) staining were performed separately on consecutive tissue sections and images were taken using vs. 120 OLYMPUS scanner. For immunohistochemistry, deparaffinized sections were labeled with anti-CD31 and anti-CD68 antibodies (Abcam) both diluted at 1:1000. Images were taken using vs. 120 OLYMPUS scanner.

#### 2.7.2. Calvaria Bone Regeneration 

A Calvaria bone procedure was performed on male Fischer 344 rats aged eight weeks (*n* = 2). Briefly, after disinfection, a linear sagittal incision was made along the top of the skull and two calvaria bone defects were created with a diameter of 6 mm on each side of the parietal bone using a trephine drill at 1500 rpm under saline irrigation. Right and left bone defects were respectively covered with samples (UV-decontaminated decellularized and devitalized Wharton’s jelly membranes) which were extended over the surrounding bone margins. Then, the periosteum and skin were carefully sutured. At weeks two and eight post-surgery, animals were imaged using micro-computed tomography scans (Skyscan 1076) with the following settings: tube voltage, 80 kV; tube current, 0.125 mA; and 35.8 μm^3^ voxel size (in vivo, 2 W) and 17.9 μm^3^ (ex vivo maintained in ethanol 70%, 8 W).

### 2.8. Statistical Analyses 

All statistical analyses were performed using GraphPad Prism software. The efficiency of decellularization was confirmed with at least six independent umbilical cords. Biochemical, physical and biomechanical characterizations were determined from at least four independent umbilical cords. For biocompatibility studies, six independent donors of Fibro and Osteo were used, and experiments were performed in duplicate. For bacterial studies, at least three independent bacterial precultures were carried out for each bacterial strain and all samples were tested for each preculture in triplicate. All results were represented as histograms (mean ± SEM). Considering the number of experiments (*n* = 6), normality tests cannot be applied. Therefore, assuming that our data did not follow a normal distribution, herein, statistical analyses were performed using a non-parametric Wilcoxon-Mann-Whitney test for independent samples. For each test, a value of *p <* 0.05 was accepted as statistically significant *p* (rejection level of the null hypothesis of equal means). 

## 3. Results and Discussion

Although widely used in clinic, the decellularization of the perinatal tissues seems an attractive option from a commercialization and regulatory approval standpoint, for the development of bioactive scaffolds. The purpose of this study sought to characterize the biological performance of decellularized Wharton’s Jelly (DC-WJ) versus devitalized Wharton’s Jelly (DV-WJ). 

### 3.1. Tissue Characterization

**Figure 1:**
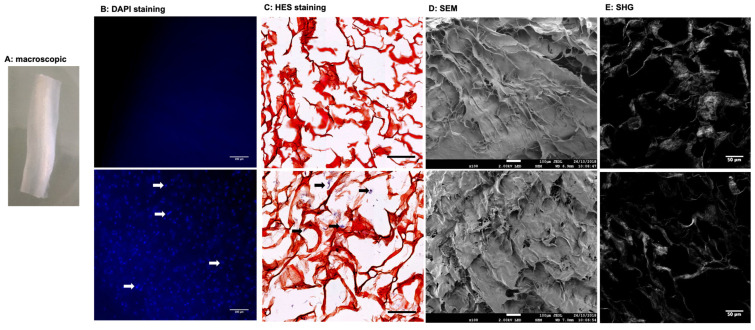
Structural features of decellularized (upper line) and devitalized (lower line) Wharton’s Jelly matrix. (**A**): Macroscopical view of DC-WJ. (**B**): Fluorescence microscopy visualization of DAPI stained nuclei (scale bar = 100 µm), (**C**): Hematoxylin-eosin-Safran (HES) staining of paraffin-embedded sections. (scale bar = 200 µm). White and black arrows highlight stained nuclei (blue and purple color, respectively). (**D**): Scanning electron microscopy (SEM) views (scale bar = 100 µm) and (**E**): Two-photon excitation laser scanning confocal microscopy and second harmonic generation (SHG) (scale bar = 50 µm).

WJ matrix is mainly composed of collagen fibers, sulfated and non-sulfated glycosaminoglycans (GAGs), and various bioactive molecules [24,25,26,27]. After removal of surrounding amniotic envelop and blood vessels, the WJ had the appearance of white jelly (Figure 1A). Histological examination confirmed that the isolated WJ matrix was rich in GAGs, collagen but also in stromal cells (Figure S1). After being proceeded, the nucleic acid moieties of stromal cells were almost fully removed as indicated by 4,6-diamidino-2-phenylin-dole (DAPI) and HES staining (Figure 1B,C) and confirmed by the DNA quantification (Table 1). These results, responding to the minimal criteria (50 ng/mg dry weight) set by Crapo and coll. satisfy the intent of the tissue decellularization [17]. Scanning electron microscopy (SEM) and Two-photons excitation laser scanning confocal microscopy and second harmonic generation (SHG) images showed a well-preserved wave-like structure of collagen fibers (Figure 1D,E). Despite a slight decrease in swelling ratio for DC-WJ (~1.3-fold decrease, *p* = 0.2), biochemical assays did not depict a significant decrease in collagen (*p* = 0.8), sulfated (*p* = 0.2) and non-sulfated (*p* = 0.9) GAGs content versus DV-WJ. Mercury intrusion porosimetry highlighted an increase in pore size from 0.1–184 to 10–184 μm for DC-WJ, increasing the surface-area-to-volume ratio and thus exchanges between the biological fluids and the WJ matrix. Such an increase could result in higher bioactivity of DC-WJ in comparison with DV-WJ [37]. Indeed, a slight increase in growth factor release and bioactive peptides was observed in the culture supernatant of DC-WJ (Table 1 and Table S1). Consistent with Jadalannagari and coll. [34], the mass spectrometry revealed that DC-WJ released in the culture medium structural proteins, including collagen alpha-3(VI) chain, collagen alpha-1(III) chain, collagen alpha-1(XII) chain III, fibronectin but also a few proteins involved in the innate immune regulation such as fibrinogen alpha chain, fibrinogen beta chain, Keratin type II and Annexin A1 (Table S1). Finally, the results of tensile tests conducted on samples in dry and wet conditions are reported in the supplementary section (Figure S2). The linear elastic modulus (EM) at high strains, being at MPa, did not show profound changes for DC-WJ in comparison with DV-WJ (*p* = 0.77 and *p* = 0.89, respectively). Taken together, these results indicated that the decellularization process had a mild effect on the structural, biochemical, physical and biomechanical properties of the native WJ (Table 1). 

### 3.2. Biocompatibility 

**Figure 2:**
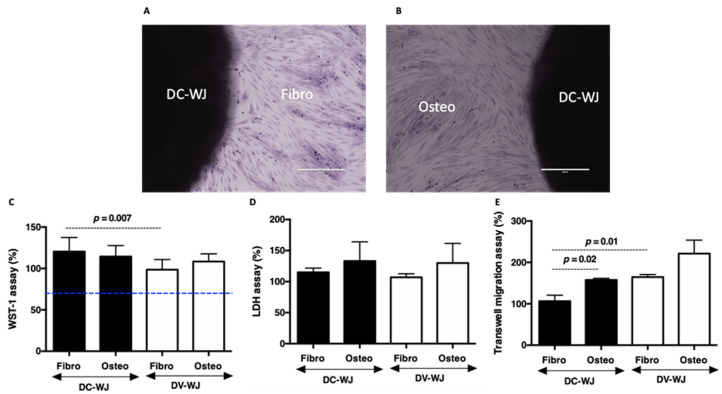
Biocompatibility of decellularized Wharton’s Jelly. (**A**,**B**): Pictures showing respectively crystal violet labelled fibroblasts (Fibro) and osteoblast (Osteo) growing in the presence of DC-WJ (scale bars = 400 µm). (**C**,**D**): Histograms reflecting percentage of cell viability and LDH release by damaged cells, respectively. Blue line indicates the threshold considered as an indicator of cytotoxic phenomenon, according to ISO standard (ISO/EN 10,993 part 5 guidelines), highlighting the absence of cytotoxic agents in both samples. (**E**): Transwell chemotaxis assay, showing a significant increase in fibroblast migration in the presence of DV-WJ compared DC-WJ (*n* = 6, Wilcoxon-Mann-Whitney test).

Decellularized allografts are classified by the regulatory agencies FDA as a human cell or tissue product and therefore do not require investigational new drug approval [11,22,38]. However, their biological cytocompatibility should be assessed prior to the product commercialization and the clinical application. In this study, the potential risks of adverse effects of DC-WJ were investigated by using primary cultured gingiva fibroblasts (Fibro) and alveolar osteoblasts (Osteo), according to ISO/EN 10,993 part 5 guideline. In addition to the important role of fibroblasts in tissue healing [35,39], the use of these primary human cells was motivated by the current clinical studies that showed a beneficial effect of WJ in the closure of defects of diabetic ulcers with osteomyelitis [30,31,32,33]. Fibro and Osteo cultured in the presence of DC-WJ and DV-WJ remained above the threshold of 70% of cell viability (versus plastic culture positive control, Figure 2A–D), threshold considered as an indicator of the cytotoxic phenomenon. Furthermore, no increase in the release of the cytosolic LDH that could result from the damaged cell membrane was observed (Figure 2C,D). Taken together, these results indicate the absence of toxic agents’ leaching from both DC-WJ (residual Triton and DNase) and DV-WJ (damaged cells). It is generally accepted that an increase in the WST-1 absorbance reflects a proliferating state of cells [38]. An increase in Fibro metabolic activity (~20%, *p <* 0.007) in the presence of DC-WJ versus control, suggests a potential effect of DC-WJ on Fibro proliferation. This increase could be partly attributed to the increase in growth factor release in the supernatant such as TGF-β (Table 1 and Table S1). These data led us to investigate the chemotactic activity of DC-WJ and DV-WJ on both cells. Chemotaxis transwell assay revealed that DC-WJ increased the Osteo migration (*p =* 0.001 vs. basal culture media) but had no effect on Fibro migration (*p =* 0.6 vs. basal culture media). In contrast, DV-WJ had chemotactic activity on both Fibro and Osteo migration (*p =* 0.0003 and *p =* 0.05 vs. basal culture media, respectively) (Figure 2E). To sum up, DC-WJ is a biocompatible and bioactive matrix favoring Fibro proliferation and Osteo recruitment, while DV-WJ showed an effect on Fibro and Osteo recruitment. 

### 3.3. Microbiological Studies 

**Figure 3:**
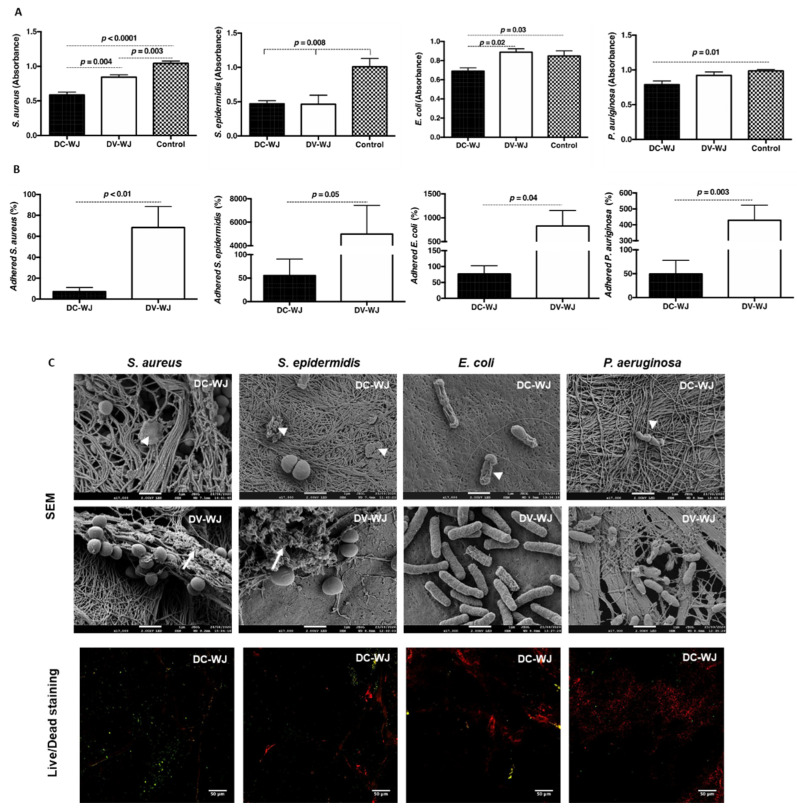
Antibacterial properties of decellularized and devitalized Wharton’s Jelly. (**A**): planktonic growth of bacteria in the presence of DC-WJ and DV-WJ. (**B**): Adhered bacteria on DC-WJ and DV-WJ. (*n* = 9, Wilcoxon-Mann-Whitney test). (**C**): Scanning electron microscopy pictures of bacteria adhered on DC-WJ (upper line) and on DV-WJ (middle line). Head arrows indicate damaged bacteria and full arrows indicate biofilm-like matrix (scale bars = 1 µm). Confocal observations of adhered bacteria on DC-WJ (lower line), in red dead bacteria and in green alive bacteria (scale bars = 50 µm).

WJ shows promising results in clinic following treatment of infected tissues [30]. The antibacterial activities of the amniotic perinatal tissue were reported previously [40,41,42] but to our knowledge, no study investigating the antibacterial properties of the WJ was reported so far. The mass spectrometry analysis showed the release from DC-WJ of antimicrobial molecules involved in the antimicrobial innate immune response but also in bacterial agglutination such as fibrinogen beta chain and Fibulin 1 (Table S1). Thus, in the following, we explored the direct effect of DC-WJ on the bacterial growth and adhesion of *Staphylococcus aureus* (*S. aureus*), *Staphylococcus epidermidis* (*S. epidermidis*), *Escherichia coli* (*E. coli*) and *Pseudomonas aeruginosa* (*P. aeruginosa*). All of these bacteria were included on the list of priority pathogens for the research and development of new antibiotics published by the World Health Organization in 2017. In comparison with the bacteria behavior in standard culture conditions, the results presented in Figure 3 showed a clear inherent effect of DV-WJ on the growth of *S. aureus* and *S. epidermidis* Gram positive strains, while no effect was noticed against *E. coli* and *P. aeruginosa* Gram negative strains. Unexpected bacteriostatic effect of DC-WJ was seen on both Gram positive and Gram negative strains as a significant reduction in the bacterial growth was demonstrated (*p <* 0.01 vs. standard culture condition) (Figure 3A). 

A multifunctional material system that kills bacteria and drives tissue healing is urgently sought in regenerative medicine. Some researchers reported that the exposure of collagen fibers in dentin increases bacterial adhesion [43]. Despite an increase in collagen exposure (i.e., depicted by SEM observations), the percentage of adhered bacteria on DC-WJ was significantly lower in comparison to DV-WJ (*p <* 0.05) (Figure 3B). Furthermore, morphological changes in bacteria shape were highlighted by SEM. Pictures showed the presence of damaged bacteria on DC-WJ while a biofilm-like matrix formed by Gram positive bacteria was observed on DV-WJ (Figure 3C). Live-dead staining and laser scanning confocal microscopy visualization confirmed the presence of propidium iodide positive bacteria (i.e., killed bacteria) on DC-WJ (Figure 3C). Due to the high fixation of propidium iodide on stromal cell nuclei, live-dead staining on DV-WJ was not shown. To sum up, DC-WJ exerts an antibacterial effect by hampering the bacteria planktonic growth but also by reducing the bacteria adhesion by several orders of magnitude compared to the DV-WJ. Herein, we thought that the antibacterial effect of the WJ matrix was not mainly due to the release of antibacterial peptides but might be attributed to the extracellular matrix and more precisely to hyaluronic acid fraction, for which the release could be increased with the increase in pore size and surface-area-to-volume ratio. Indeed, the local application of hyaluronic-based compounds has been demonstrated to be protective against bacterial proliferation, even if studies in this context are few [44,45]. Furthermore, due to their antiadhesive and antifouling properties, hyaluronic acid and its composites are considered as an attractive, non-antibiotic, option to minimize the impact of biofilm-related infections [46,47]. However, in this study, we cannot exclude the presence of other antibacterial agents within the WJ matrix. A deeper analysis of the WJ composition using proteomic and peptidomic approaches is thus required.

### 3.4. Acute Inflammatory Response

**Figure 4:**
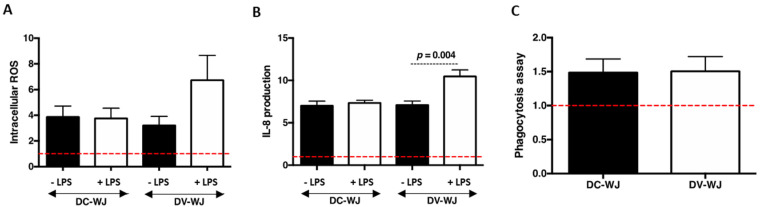
Innate neutrophil responses to decellularized and devitalized Wharton’s Jelly. (**A**,**B**): Intracellular accumulation of ROS in neutrophils and IL-8 production by neutrophils in the presence of DC-WJ and DV-WJ. (**C**): Median fluorescence intensity of phagocyted Bioparticles. All the results were normalized to the basal production of neutrophils indicated by red dashed lines; *n* = 4, Wilcoxon-Mann-Whitney test.

Allogeneic WJ has been used as a dressing to cover chronic wounds [30,31,32,33]. Despite the beneficial properties that have been reported, the immune privilege of the umbilical cord is thought to be linked to the poorly vascularized site. Innate immune defense mechanisms are in charge of protecting the host from foreign threats. They orchestrate debridement, remodeling and ultimately, return to normal homeostasis of compromised tissue. Inflammatory cells are a fulcrum in tissue healing, producing enzymes, cytokines and unstable reactive oxygen species (ROS) to direct the dynamics of tissue healing and biomaterial integration. The decellularization procedure led to the release of extracellular matrix molecules (i.e., collagen, fibronectin, fibrinogen…, Table S1), that could be converted into a “danger signal”, leading to an undesired inflammatory response after implantation [48]. Thus, in the following, we investigated the innate immune response to DC-WJ and DV-WJ. Neutrophils, the first line of defense towards foreign bodies, are pivotal in deciding the fate of implanted materials [35,36]. Therefore, herein, leukocyte respiratory burst response (i.e., intracellular accumulation of ROS), IL-8 production and phagocytosis assay were performed on human neutrophils in the presence of DC-WJ and DV-WJ. Focusing the current study on the neutrophil responses to the WJ samples, the following results were normalized to the basal production of neutrophils, avoiding differences in neutrophil response between individuals. Flow cytometry and ELISA analyses indicated an increase in intracellular accumulation of ROS and in IL-8 release by neutrophils following the contact with both DC-WJ and DV-WJ (~5- fold increase; *p <* 0.0001), while no marked effect of samples on phagocytosis capability was noticed (Figure 4A–C). Despite the release of bioactive molecules from DC-WJ, we can conclude that the neutrophil activation in the presence of both DC-WJ and DV-WJ are of comparable magnitude. Thus, neither the use of Triton ×100 and DNase during the processing nor the presence of residual DNA and cellular components in DV-WJ affects the neutrophil response to the tissue. Recently Gupta et al., identified the presence of mediators such as intercellular adhesion molecule-1 and monocyte chemotactic protein-1 in WJ matrix [20]. Involved in leukocyte accumulation which are required for wound healing, these mediators associated with others could be responsible for the increased production of ROS and IL-8 by neutrophil in the presence of WJ. In the second time, the synergistic effect of DC-WJ and DV-WJ and lipopolysaccharide (LPS) inflammatory stimulus was assessed. In contrast to DV-WJ, no cumulative effect of LPS and DC-WJ on the intracellular accumulation of ROS and the release of IL-8 was observed, suggesting a quenching of LPS effect on neutrophil activation by the DC-WJ itself. 

**Figure 5:**
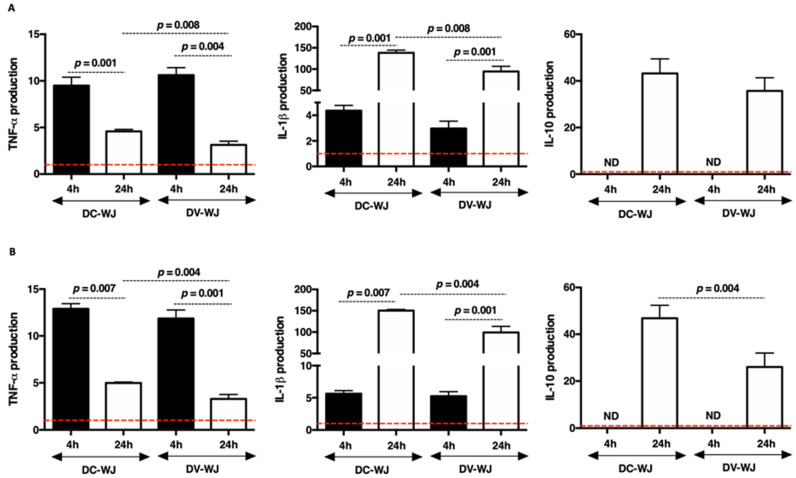
Innate monocyte responses to decellularized and devitalized Wharton’s Jelly. (**A**): cytokine production following contact with DC-WJ and DV-WJ. (**B**): cytokine production following contact with DC-WJ and DV-WJ co-stimulated with LPS. Results were normalized to the basal monocyte production indicated by the red dashed lines; *n* = 6, Wilcoxon–Mann–Whitney test.

After graft implantation, the host’s monocytes are attracted to the site and differentiated into macrophages, responding secondly to the biomaterial [35,36]. The macrophage activities are closely related to immune responses, inflammation, foreign body responses and biodegradation of materials. Therefore, macrophages are essential for the regeneration process which occurs via the secretion of cytokines and the regulation of angiogenesis. In this study, we sought to investigate the acute monocyte response to DC-WJ. For that, human peripheral CD14 positive monocytes were cultured in direct contact with both DC-WJ and DV-WJ. Cytokines data were obtained from culture supernatants after 4 h and 24 h and normalized to the basal cytokine production. In comparison with basal cytokine production, the ELISA results showed that DC-WJ and DV-WJ activated the production of TNF-α and IL-1β pro-inflammatory cytokines but also the production of IL-10 anti-inflammatory cytokine. In comparison with 4 h, 24 h of contact showed a decrease in TNF- α (*p <* 0.004) while an increase in IL-1β (*p <* 0.001) release was seen. IL-10 was detected only in 24 h harvested supernatant (Figure 5A). Regarding the proinflammatory cytokines, the monocyte activation after 24 h by DC-WJ was in higher magnitude than DV-WJ (~1.5 fold; *p =* 0.0008). The same experiment was conducted in the presence of an LPS stimulus. The addition of LPS stimulus activates the monocyte in the same way as without LPS. Compared to DV-WJ/LPS co-stimulation, DC-WJ/LPS co-stimulation increased the production of TNF-α, IL-1β and IL-10 (>1.8-fold increase, *p <* 0.004) by monocytes after 24 h of contact (Figure 5B). Taken together, our results suggest that neither DC-WJ nor DV-WJ and LPS co-stimuli showed a switch in monocyte response in comparison with DC-WJ and DV-WJ stimuli. 

Proper graft and biomaterial integration depend on cellular and fibrovascular ingrowth, followed by tissue remodeling. Biomaterial that induces elevated IL-1β production by monocytes may allow the host’s tissue integration via fibroblast proliferation [49,50,51]. To sum up, the acute monocyte profile seemed predictive of a moderate inflammatory response towards DC-WJ and DV-WJ following the implantation. Furthermore, the biocompatibility of the DC-WJ was confirmed since no increasing deleterious effect on monocyte activation was highlighted.

### 3.5. Immunomodulatory Response 

**Figure 6:**
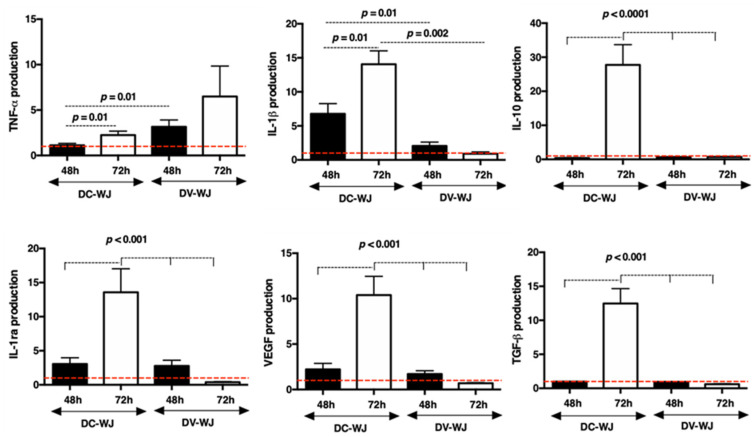
Macrophage polarization in the presence of decellularized and devitalized Wharton’s Jelly. Cytokine production following contact with DC- and DV-WJ. Results were normalized to DNA and M0 production, indicated by the red dashed lines; *n* = 4, Wilcoxon-Mann-Whitney test.

Over the last several years, two subpopulations of macrophages have been described (i) M1 phenotype, which favors a pro-inflammatory state, and (ii) M2 phenotype, which favors wound healing and tissue remodeling [52,53]. Chemically treated tissues were associated with an M1 macrophage-driven response, resulting in chronic inflammation and scar formation [49,54]. As described previously, DC-WJ is a bioactive matrix, able to release in the extracellular medium immunoregulatory mediators (Table S1). Herein, we investigated the immunomodulatory action of DC-WJ and DV-WJ on macrophage polarization. For that, THP-differentiated macrophage (M0) were cultured in direct contact with both samples and the cytokine production was analyzed at 48 h and 72 h post-culture. The presented data were normalized to DNA content and M0 basal production (Figure 6). This study revealed that macrophages after 72 h of contact with DC-WJ over-produced the pro-inflammatory (IL-1β and TNF-α, *p =* 0.001) but also the anti-inflammatory mediators (VEGF, IL-1ra, IL-10 and TGF-β, *p <* 0.001). On the contrary, in the presence of DV-WJ, the secretion of proinflammatory mediators remained dominant as no over-production of IL-1ra, VEGF, TGF-β and IL-10 was noticed. Interestingly, DC-WJ stimulated the production of IL-1 β at 72 h but also IL-1RA, a specific interleukin-1 receptor antagonist that competitively binds the IL-1 receptor, thereby blocking the IL-1-mediated cellular effects [55]. An appropriate immune response could promote tissue regeneration, by contrast, excessive and over inhibited one might hamper tissue healing. It was described that the effective and timely switch from an M1 phenotype to an M2 phenotype is four to seven days post-implantation [52,53]. These results thus indicate that macrophage effectively modulated their phenotypes and polarization states from the resting M0 phenotype to the hybrid M1 and M2 phenotype in the presence of DC-WJ. These results are consistent with Keane et al. who reported that a reduction in DNA content in the tissue helps the tissue to promote the macrophage phenotype to M2 in vitro [12]. Furthermore, in addition to the increase in specific exchange area, the mass spectrometry analysis suggested that the effect of DC-WJ on macrophage polarization towards the hybrid M1 and M2 phenotype could be attributed to the release of matrix derivative molecules (i.e., collagen alpha-3(VI) chain, collagen alpha-1(III) chain and collagen alpha-1(XII) chain III, fibronectin and GAGs) [48,56]. 

### 3.6. In Vivo Responses

**Figure 7:**
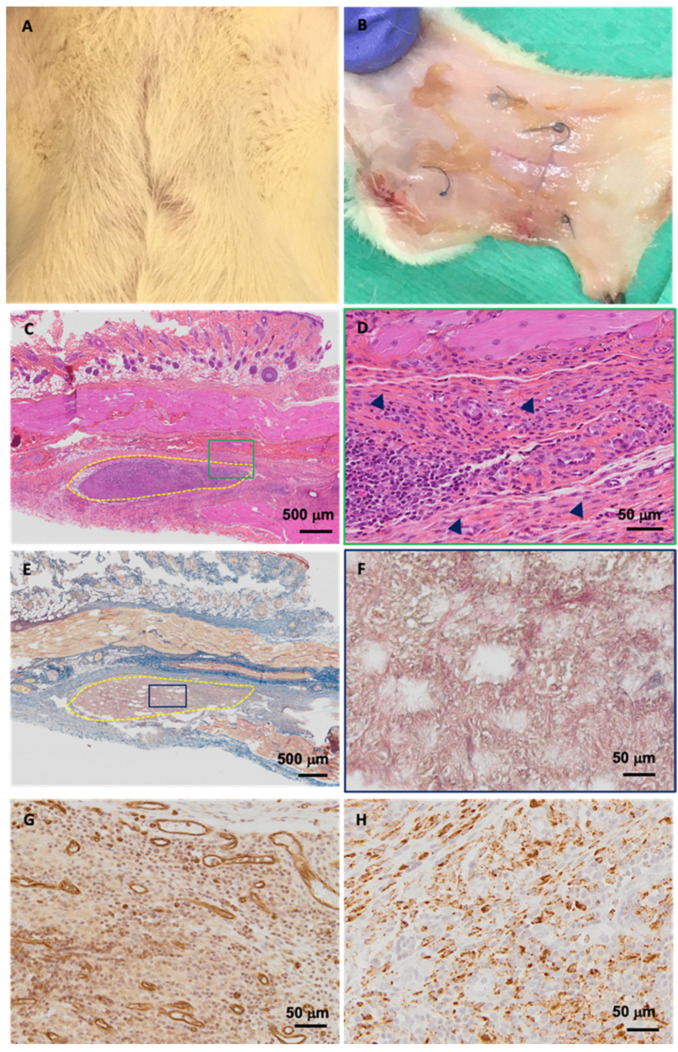
Subcutaneous biocompatibility of decellularized Wharton’s Jelly. (**A**,**B**): Macroscopical examination of the wound recovery and explanted tissue, respectively. (**C**,**D**): Hematoxylin-eosin-Safran (HES) staining of paraffin-embedded explant (delimited by dashed yellow line) at lower and higher magnifications, respectively. Head arrow point fibroblasts. (**E**,**F**): Masson trichrome staining of paraffin-embedded explant at lower and higher magnifications, respectively. Higher magnifications correspond to green and blue rectangles. (**G**): CD31 immunohistochemical staining and (**H**): CD68 immunohistochemical staining of the granulation tissue. (scale bar = 500 and 50 µm).

Biocompatibility is the “ability of a material to perform with an appropriate response host in a specific application”. Overall, the above-described data suggest that the production of cytokines is likely predictive of a moderate inflammatory host’s response to DC-WJ, favoring wound healing and tissue remodeling. However, these resulting data used isolated populations of mononuclear cells, which removed the potential for inter-leukocyte signaling. To confirm the bio-relevance use of DC-WJ, they were implanted in immunocompetent rats, in order to be able to take immunological reactions into consideration. The first experiment was conducted subcutaneously for three weeks (chronic inflammation phase). All animals had a good recovery and no animals showed pain after surgery. Healing of the skin incisions created for subcutaneous graft implantation occurred without any wound complications or macroscopically visible signs of inflammation and edema. Xenografts (i.e., human WJ) were tolerated by immunocompetent rats as a preserved dermal integrity, with intact hair follicles were observed (Figure 7A,B). The histo-morphological examination of the explanted DC-WJ showed neither the formation of a thick fibrous capsule nor multinucleated giant cells within and around the implanted graft, which usually emerge as a severe immunological response to the transplantation of foreign matter [52,53]. The data presented herein contrasts previously published work in which collagen-based material showed an excessive foreign body reaction with a formation of a thick capsule and giant multi-nucleated cells [57], but are consistent with Bullard and coll., who demonstrated total resorption of devitalized umbilical cord without capsule formation [58]. HES staining revealed the presence of a granulation tissue typical of a normal wound healing response around the DC-WJ explant without fibrosis inside it (Figure 7C,D). Masson’s trichrome confirmed the absence of collagen fiber, a predominant part of WJ matrix, suggesting total resorption of the DC-WJ which was replaced with an inflammatory resorptive tissue (Figure 7E,F). Despite the absence of fibroblast recruitment during the in vitro chemotaxis assay, the infiltration of spindle-shaped fibroblast-*like* in collagen tissue was identified at the periphery of the granulation tissue (Figure 7D head arrows). Finally, immunohistochemistry revealed the presence of CD31 corresponding to blood vessels and CD68 positive macrophages, suggesting a moderate inflammation (Figure 7G,H). Hence, we can conclude that DC-WJ is biocompatible and well tolerated from the host’s organism.

**Figure 8:**
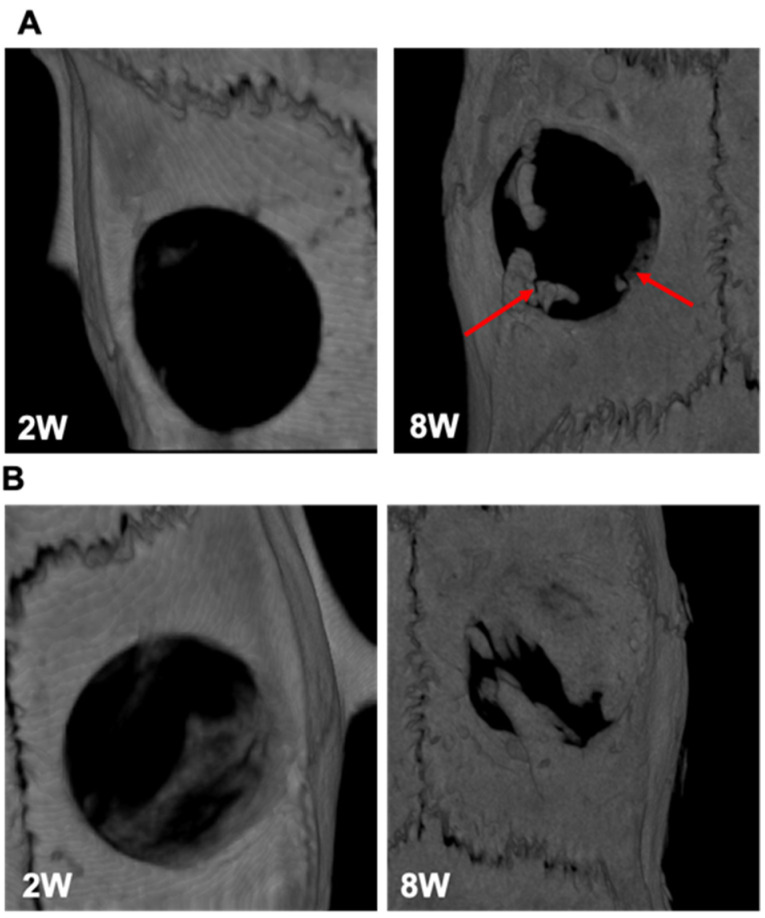
Calvaria bone defect regeneration. (**A**,**B**): MicroCT results following implantation of DC-WJ and DV-WJ, respectively. The two weeks (2 W) scans were obtained in vivo (35.8 µm) while eight weeks (8 W) scans were obtained ex vivo (17.9 µm). Red arrows indicate marginal bone regeneration in the presence of DC-WJ.

It is well known that neovascularization is a positive response to the introduction of a foreign body in an organism, which ensures a proper supply of biochemical signals, oxygen and nutrients to the cells of the tissue surrounding the implant [59]. Our observations are consistent with Ungerleider and coll., who used WJ-derived hydrogels as a neovascularization therapeutic approach to increase the perfusion in a hindlimb ischemia model [60]. An increase in neo-vascularization without amplifying inflammation preludes the suitable biocompatibility of DC-WJ for the bone regenerative procedure [61]. The second preliminary experiment, conducted in calvaria bone defect (*n* = 2), sought to analyze the temporal evolution of the bone following the implantation of the DC-WJ membrane. In guided bone regeneration, a collagenous barrier membrane is applied in order to prevent soft tissue invasion into the bone defect, allowing sufficient time and space to guide the growth of bone-forming cells into defects [62]. Contrasting with the in vitro chemotaxis assay, representative micro-CT results of rat calvarias covered with DC-WJ membrane at weeks two and eight are displayed in Figure 8A. Very little bone regeneration evidence could be found at the marginal area of the calvarial defect. Immunomodulation is a ‘double-edged sword’, and different extents of immunomodulation lead down different paths. Osteoimmunomodulation is emerging to support a broader role of M1 macrophages in bone healing and regeneration, while M2 macrophages showed pro-fibrotic effects, promoting macrophages fusion into foreign body giant cells [57,63]. Compared to Bio-Gide^®^ membrane, the clinical gold standard, which elicits M1 macrophages [57], and DV-WJ (Figure 8B), herein, we thought that the hybrid immunomodulatory behavior renders the DC-WJ not suitable with guided bone regeneration field. We cannot exclude that the limited bone regeneration could be also due to lack of sufficient mechanical strength and collapse into bone defect area [64].

## 4. Conclusions

Herein, we demonstrated the superiority in the use of decellularized Wharton’s Jelly matrix in comparison with devitalized Wharton’s Jelly as an engineered biomaterial. It was demonstrated that the DC-WJ matrix is in accordance with the established criteria for decellularized tissues to avoid cell and host adverse reactions. Although the decellularization did not deeply affect the structural, biochemical and biomechanical cues of the matrix, the increase in the bioactivity following the decellularization increased its antibacterial and immunomodulatory properties crucial for tissue healing. These results support the hypothesis of the potential use of DC-WJ as a biomaterial for tissue regeneration applications, particularly when a membrane is needed to separate tissues, organs or other biologic compartments. In large-scale future studies, the investigation of the potential use of decellularized Wharton’s Jelly in soft tissue regeneration might promote their faster and safer translation into clinical practice.

## Figures and Tables

**Table 1:** Biochemical, physical and biomechanical characterization of decellularized and devitalized Wharton’s Jelly (*n* = 5).

	DC-WJ	DV-WJ	*p*
DNA content (ng/mg of the dry tissue)	Below the limit detection	664.12 ± 210.75	0.007
Collagen (mg/mg of dry tissue)	0.88 ± 0.09	0.89 ± 0.06	0.8
Sulfated GAGs (μg/mg of dry tissue)	3.14 ± 1.4	1.6 ± 0.6	0.2
Non sulfated GAGs (μg/mg of dry tissue)	15.08 ± 3.4	15.11 ± 1.9	0.9
Released VEGF (pg/mL/mg of dry tissue)	126 ± 17	37 ± 17	0.01
Released HGF (pg/mL/mg of dry tissue)	451 ± 148	296 ± 54	0.16
Released TGF-β (pg/mL/mg of dry tissue)	445 ± 25	436 ± 24	0.67
Porosity range (μm)	10 to 184	0.1 to 184	-
Total porosity (%)	~79		-
Swelling ratio (g/g)	14.0 ± 6.7	18.5 ± 9.2	0.2
Linear elastic modulus (dry condition) (MPa)	12.69 ± 3.87	12.57 ± 3.08	0.77
Linear elastic modulus (wet condition) (MPa)	0.64 ± 0.22	0.61 ± 0.11	0.89

(VEGF: vascular endothelial growth factor, HGF: hepatic growth factor, TGF-β: Transforming growth factor beta). *p* values from Wilcoxon-Mann-Whitney test.

## Data Availability

The data that support the findings of this study are available from the corresponding author upon reasonable request.

## Supplementary Materials

The following supporting information can be downloaded at: https://www.mdpi.com/article/10.3390/biomedicines10020227/s1, Figure S1: Histological characterization of native Wharton’s Jelly, Figure S2: Mechanical characterization of decellularized and devitalized Wharton’s Jelly, Table S1: Proteins identified in DC-WJ supernatant (serum free-culture medium) by mass spectrometry. Reference [65] are cited in the supplementary materials.
