# Supplementary material for: Antibacterial and Immunomodulatory Properties of Acellular Wharton’s Jelly Matrix
Source: Biomedicines. 2022 Jan 21;10(2):227. doi: 10.3390/biomedicines10020227 (PMC8869352; doi:10.3390/biomedicines10020227)
Supplement: Supplementary file 1 [file biomedicines-10-00227-s001.zip › biomedicines-1525127-supplementary.pdf]

Antibacterial and immunomodulatory properties of acellular Wharton's jelly matrix.

Materiel and methods

1. Mechanical properties

The mechanical properties of samples were tested through quasi static tensile tests up to failure. The loading sequence was divided into two steps: a) a dry test under elastic limits with an imposed strain around 1.6 % to avoid any damages followed by b) a PBS hydrated at 37 °C one allowing a full mechanical response characterization. In between both steps, five minutes were given for the sample to accommodate prior to be tested with 1.6 % strain loads and eventually up to failure. All loadings were performed at a 0.01 $mm.s^{-1}$ velocity to remain in the quasi static framework. A Universal Testing Machine Zwicky 0.5 equipped with a 10 N loadcell was used to measure samples' response. The engineering stress and strain definition was used to process the force displacement curves and measure the linear elastic moduli.

2. Mass spectrometry

Freeze-dried samples were soaked in 1 mL of fetal bovine serum (FBS) free α-MEM (Lonza, Saint Quentin, France) for 72 h and the released macromolecules were analyses by mass spectrometry. Each supernatant was precipitated with DOC/TCA and resuspended in 50 µL Urea, 6M, Tris, 50 mM, pH 8.0. From these, 20 µL were processed as follows: Cysteine residues were reduced by addition of 4 mM DTT for 45 min, alkylated by addition of IAA at 40 mM for another 45 min and IAA was blocked by addition of another 40 mM DTT for 10 min. 180 µL Tris, 50 mM, $CaCl_2$, 1 mM were added together with 100 ng sequencing grade trypsin and digestion was allowed overnight at 37 °C. Samples were acidified by addition of 10 µL TFA 10 %. Samples were fractionated by nanoHPLC on an Ultimate3000 system equipped with a 20 µL sample loop, a pepMap 100 C18 desalting precolumn and a 15 cm pepMap RSLC C18 fractionation column (all from Dionex, Villebon-sur-Yvette, France). Samples (6.4 µL) were injected using the µl pickup mode and eluted by a 2 to 45 % ACN gradient over

60 min at 300 nL/min. Fractions (340, 9 seconds each) were collected on a ProteineerFcII (Bruker) over 51 min and eluted fractions were directly mixed on MTP-1536 BC target (Bruker) spots to α-cyano-4-hydroxycinnamic acid (Bruker). LC-MALDI runs were processed using dedicated automatic methods piloted by WARP-LC software on an Autoflex speed MALDI-TOF/TOF mass spectrometer (Bruker), first in MS mode only, in the 700-3500 mass range, using next-neighbour external calibration for all MALDI spots. MS runs were used for label-free relative quantification strictly as described[65]. On this basis, masses found significantly changed between experimental groups were selected for MS/MS analysis in LIFT mode. Thereafter, all masses with S/N > 6 from one single run were also processed for LIFT fragmentation and all resulting fragmentation data were used for database interrogation. Peptide assignments were performed from TOF/TOF spectra by Mascot interrogation (Matrix Science) of the Swissprot human proteome database piloted in Mascot (Matrix Science) and compiled by Proteinscape (Bruker) with a mass tolerance of 50 ppm in TOF mode and 0.8 Da in TOF/TOF mode, with optional cysteine carbamidomethylation, methionine oxidation and with trypsin cut allowing one mis-cleavage. The minimal mascot score for peptides was set to 20 and that for proteins was set to 80. Protein change calculations and statistics were also performed strictly as previously[65]. Identification results were cross-validated by interrogating an irrelevant database using the same criteria.

3. Histology

DC- and DV-WJ samples were embedded in paraffin and cut into 4 μm sections (rotation microtome RM2055, Leica Microsystems, Nanterre, France). Hematoxylin-Eosin-Saffron (HES), Masson Trichrome (MT) and alcian blue staining were performed and images were taken using VS 120 OLYMPUS scanner.

Results

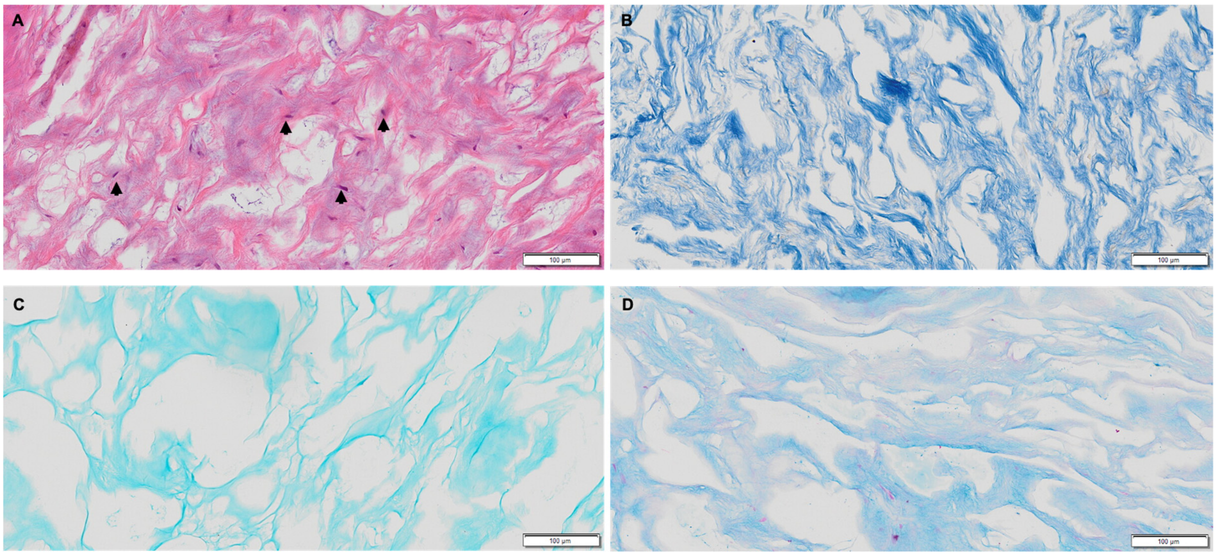

Figure S1. Histological characterization of native Wharton's Jelly. Paraffin- stained section with (**A**): Hematoxylin-eosin-Safran (HES), (**B**): Masson trichrome, (**C**) and (**D**): Alcian blue staining at pH 2.5 and 1.5, respectively (scale bar = 100 µm). Head arrows highlighted stained nuclei.

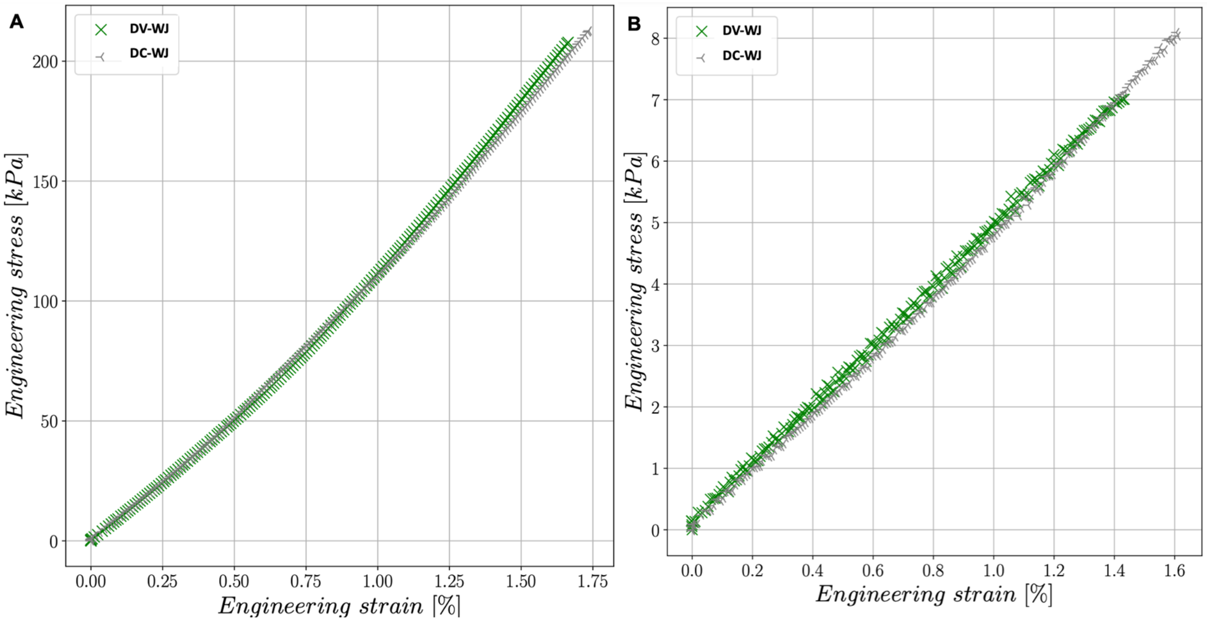

Figure S2: Mechanical characterization of decellularized and devitalized Wharton's Jelly. (**A**): dry and (**B**): wet conditions.

Table S1: Proteins identified in DC-WJ supernatant (serum free-culture medium) by mass spectrometry.

Protein	Location	Molecular Weight (KDa)	Sequence Coverage (%)
Collagen alpha-3(VI) chain	Extracellular matrix	343.5	5.9
Transforming growth factor-beta-induced protein ig-h3	Extracellular matrix	74.63	22.5
Fibrinogen beta chain	Extracellular matrix	55.9	24
Collagen alpha-1(XII) chain	Extracellular matrix	332.9	6.5
Tenascin	Extracellular matrix	240.7	4.7
Fibrinogen gamma	Extracellular matrix	55.1	19.2
Collagen alpha-1(III) chain	Extracellular matrix	138.5	8.2
Periostin	Extracellular matrix	93.3	4.4
Fibronectin	Extracellular matrix	262.5	2.3
Serum albumin	Extracellular matrix	69.3	8.4
Fibrinogen alpha chain	Extracellular matrix	94.9	2.5
Fibulin-1	Extracellular matrix	77.2	5
Annexin A1	Extracellular matrix	38.7	4.6
Lumican	Extracellular matrix	38.4	6.5
Keratin, type I cytoskeletal 13	Intracellular	49.55	10.5
Keratin, type I cytoskeletal 9	Intracellular	62	3.7
Keratin, type I cytoskeletal 19	Intracellular	44.1	12
Keratin, type I cytoskeletal 10	Intracellular	58.8	9.2
Keratin, type II cytoskeletal 4	Intracellular	57.2	2.6
Ig kappa chain C	Cellular membrane	11.6	15.1
